# Anisotropic SmFe_10_V_2_ Bulk Magnets with Enhanced Coercivity via Ball Milling Process

**DOI:** 10.3390/nano14161329

**Published:** 2024-08-08

**Authors:** Tian Hong Zhou, Baochao Zhang, Xing Zheng, Youngwoon Song, Pingzhan Si, Chul-Jin Choi, Young-Rae Cho, Jihoon Park

**Affiliations:** 1 Nano Materials Research Division, Korea Institute of Materials Science, Changwon 51508, Republic of Korea; zhouth@kims.re.kr (T.H.Z.);; 2 Division of Materials Science and Engineering, Pusan National University, Busan 46242, Republic of Korea

**Keywords:** magnetic properties, ThMn_12_, grain boundary, microstructure, ball milling

## Abstract

Anisotropic bulk magnets of ThMn_12_-type SmFe_10_V_2_ with a high coercivity (*H_c_*) were successfully fabricated. Powders with varying particle sizes were prepared using the ball milling process, where the particle size was controlled with milling time. A decrease in *H_c_* occurred in the heat-treated bulk pressed from large-sized powders, while heavy oxidation excessively occurred in small powders, leading to the decomposition of the SmFe_10_V_2_ (1–12) phase. The highest *H_c_* of 8.9 kOe was achieved with powders ball-milled for 5 h due to the formation of the grain boundary phase. To improve the maximum energy product ((*BH*)*_max_*), which is only 2.15 MGOe in the isotropic bulk, anisotropic bulks were prepared using the same powders. The easy alignment direction, confirmed by XRD and EBSD measurements, was <002>. Significant enhancements were observed, with saturation magnetization (*M_s_*) increasing from 59 to 79 emu/g and a remanence ratio (*M_r_*/*M_s_*) of 83.7%. (*BH*)*_max_* reaching 7.85 MGOe. For further improvement of magnetic properties, controlling oxidation is essential to form a uniform grain boundary phase and achieve perfect alignment with small grain size.

## 1. Introduction

The SmFe_12_-based compounds with a tetragonal ThMn_12_ structure (space group *I4/mmm*) are considered to be promising candidates for new high-performance permanent magnets due to their intrinsic magnetic properties, high Fe content, and the relatively inexpensive rare-earth element Sm [1,2]. It possesses higher theoretical magnetic performance compared to Nd-Fe-B sintered magnets. For example, the Sm(Fe_0.8_Co_0.2_)_12_ compound with the ThMn_12_-type structure has been realized in thin films, achieving a high *M_s_* of 1.78 T and a high anisotropy field of 12 T [3]. However, there are no reports of SmFe_12_-binary compounds with high performance in the bulk magnet. Two main reasons account for this: Firstly, the SmFe_12_-binary phase is unstable and can only be produced in thin films. To form a stable SmFe_12−x_M_x_ phase, partial substitution of Fe with other stabilizing elements, such as Ti, V, Cr, Mo, W, Al, Si, and Ga, is necessary [4]. Second is the low *H_c_* in the Sm(Fe_0.8_Co_0.2_)_12_ compound, which impedes the development of SmFe_12_-based bulk magnets [5].

In order to obtain stable bulk magnets with high coercivity *H_c_*, researchers have developed various methods to modify the microstructure. One effective approach is to decrease the grain size to the single domain size, which has been proven to enhance magnetic performance significantly. A common method for achieving nano-scale grain sizes is the melt spinning technique. There are many reports of obtaining the ThMn_12_-type magnets with good performance [6,7,8,9]. The grain size can be controlled by heating the amorphous ribbons or by melt-spinning at specific speeds. Qian et al. [10] successfully produced high-density Sm(Fe_0.8_Co_0.2_)_11_Ti bulk magnets with average grain sizes ranging from 30 to 74 nm. An average grain size of 30 nm was achieved in melt-spun ribbons with enhanced *H_c_*, as reported by Zhao et al. [11]. Additionally, the formation of a non-ferromagnetic grain boundary phase (GBP) is effective in improving *H_c_* by providing magnetic decoupling among the main magnetic phase and hindering the movement of the domain wall. Elements such as Cu, Ga, B, and V have been found beneficial in forming the GBP in SmFe_12_-based magnets [8,12,13,14]. We successfully formed a Sm-Cu-Ga-rich GBP in heat-treated bulks with nano-scale grain sizes, resulting in enhanced *H_c_* [14]. Liu et al. reported achieving a *H_c_* of 6 kOe in Sm(Fe_0.8_Co_0.2_)_11_TiB_0.25_ melt-spun ribbons with an average grain size of 150 nm [8]. However, since an amorphous phase was obtained as an intermediate during this process, it is challenging to achieve oriented bulk magnets, which are crucial for improving (*BH*)*_max_*. Unlike Nd-Fe-B magnets, the hot-deformation process has been found to be ineffective in aligning SmFe_12_-based bulks [15,16]. In V-substituted SmFe_12_-based compounds, high *H_c_* can be obtained even with a large grain size. High *H_c_* Sm-Fe-V-based oriented bulk magnets have been achieved with *H_c_* above 10 kOe by forming the Sm-rich GBP after hot-compacting jet-milled powders with a grain size of several microns [17,18].

In our previous work, high *H_c_* was successfully achieved in fully dense SmFe_10_V_2_ isotropic bulk magnets using the jet-milling process [19]. However, the magnetically aligned green bodies were too weak to retain their shape due to the good sphericity of the jet-milled powders. This resulted in a failure to press and sinter anisotropic bulks, as well as a high loss of powders during the jet-milling process. In this study, we used a ball milling process to prepare SmFe_10_V_2_ powders. It is well known that ball-milled powders have an irregular shape, and the powder loss during this process is negligible. We investigated the influence of powder size, controlled by milling times, on the microstructure and magnetic properties. Subsequently, we prepared oriented bulks to study the impact of microstructure on their magnetic properties.

## 2. Experiments

The SmFe_10_V_2_ ingots were synthesized through arc-melting using high-purity Sm (99.9%), Fe (99.95%), and V (99.9%) pieces. An additional Sm was introduced to compensate for Sm loss due to evaporation during the fabrication process and to facilitate the formation of a Sm-rich GBP. The following homogenization process was carried out at 1000 °C for 20 h under an Ar atmosphere. Subsequently, the homogenized ingots were crushed through hydrogen decrepitation at 250 °C for 5 h and manually ground into powders below 150 μm in size. The low-energy ball milling process was then used to further reduce the particle size. ZrO_2_ milling balls with a size of 5 mm were used during the milling process. The weight ratio of the milling balls and powders is 20:1. Milling times ranged from 3 to 50 h to produce particles of different sizes, allowing the investigation of their influence on the microstructure and magnetic properties of the final heat-treated bulks. The 1.5 g as-milled powders (BM3–BM50) were pressed under 0.4 GPa using a stainless steel mold to obtain round, low-density green bodies with a diameter of 10 mm. Then, the green bodies were pressed again under 3.5 GPa, restricted by the same-thickness iron rings, to form high-density green bodies, followed by heat treatment at 1140 °C for 30 min under an Ar atmosphere. The heat-treated bulks were named from BM3-HT to BM50-HT depending on the milling time, as shown in Table 1. Furthermore, in order to improve the magnetic performance, the powders were initially pressed under a 10 kOe magnetic field with a pressure of 0.4 GPa and then pressed again under higher pressures. The powders inside the green bodies were arranged using the external magnetic field to obtain magnetic anisotropic bulks. The influence of pressure on the anisotropic bulks was investigated using stepped high pressures of 0.8, 1.0, 1.5, 2.6, 3.5, and 4.0 GPa. Finally, the anisotropic green bodies were heat-treated under the same conditions as the isotropic ones. Table 1 shows the sample names to distinguish all the samples during the experiment.

The phase and crystalline structure were measured using X-ray diffraction (XRD, D/Max-2500VL/PC, Rigaku, Tokyo, Japan) analysis with Cu-Kα (λ = 1.5406 Å) radiation. The magnetic properties of the bulk samples were examined using a vibrating sample magnetometer (VSM, MicroSense EZ9, KLA, Santa Clara, CA, USA), which can provide a maximum magnetic field of 27.5 kOe with a sample space of 10 mm at room temperature. Square samples weighing 20–100 mg, with side lengths smaller than 3 mm, were measured in a magnetic field ranging from −25 kOe to 25 kOe to obtain the hysteresis loops. Specimen density was measured with the Mettler Toledo Balance XPE205 using the Archimedes method. Microstructures were observed using scanning electron microscopy (SEM, JSM-6610LV, JEOL Ltd., Tokyo, Japan) and field emission SEM (FE-SEM, JSM-7800F, JEOL Ltd., Tokyo, Japan). Electron backscatter diffraction (EBSD, Oxford, Symmetry, JEOL Ltd., Tokyo, Japan) in FE-SEM (JSM-7800F) was used to investigate the crystal orientation of the 1–12 grains in the anisotropic bulks with a step size of 0.2 μm. The tetragonal SmFe_10_V_2_ structure was used to index during the measurement.

## 3. Experimental Results and Discussion

### 3.1. Microstructures and Magnetic Properties of the Ball-Milled Powders

**Figure 1:**
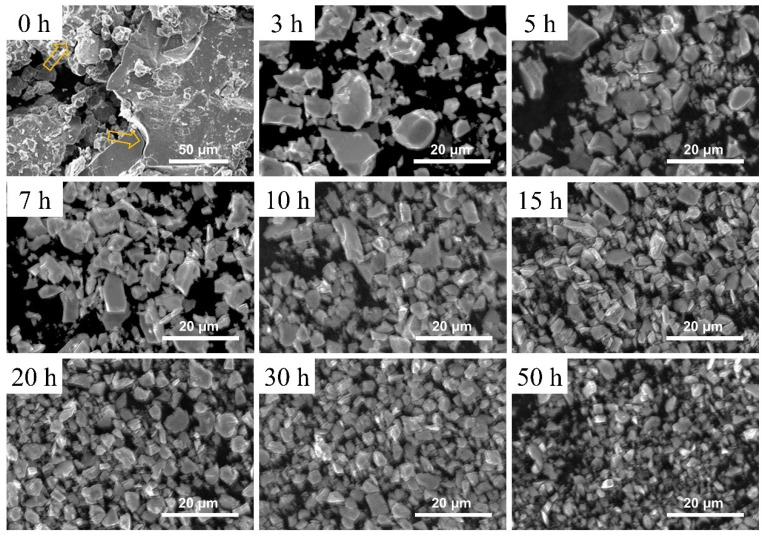
SEM images of the H_2_ decrepitated powder (0 h) and the ball-milled powders with different ball milling times from 3 to 50 h (BM3–BM50). The yellow arrows show the intergranular fracture made by the H_2_ decrepitation. The powder size distribution becomes finer with longer milling times, with maximum and minimum sizes varying severalfold within the same milling time.

**Figure 2:**
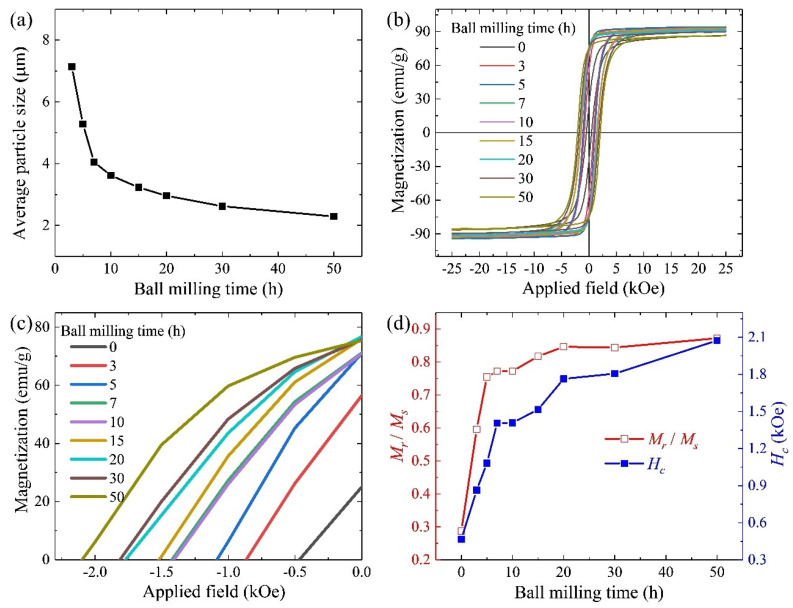
Average size (**a**), hysteresis loops (**b**,**c**), and the tendency of *M_r_*/*M_s_* and *H_c_* (**d**) of the H_2_ decrepitated powder (0 h) and the ball-milled powders with different ball milling times, from 3 to 50 h (BM3–BM50).

The H_2_ decrepitated powder and the ball-milled powders with different milling times, ranging from 3 to 50 h, were analyzed using SEM, as shown in Figure 1. After grinding the H_2_ decrepitated ingots, the powders exhibited a wide range of sizes, from several microns to 100 microns. The powder surface was smooth. There were some winding crucks in the large-size powders. The fractures made by H_2_ decrepitation on the 1–12 grain boundaries limited the formation of multi-grain particles after the ball milling process. The powder size decreased with longer milling times. The maximum and minimum sizes of the powders with the same ball milling time varied significantly. The detailed distribution of powder size is shown in Figure S1 in the Supplementary File. The size of the powders in the same sample varies widely, ranging from less than 1 μm to 20 μm. The average powder size decreased as the ball milling time increased. A summary of the powder sizes is shown in Figure 2a according to Figure S1. Initially, the powders sharply decreased in size to nearly 3.5 μm. From 10 to 50 h of milling, there was a slight further decrease in powder size. According to Figure S2 in the Supplementary File, the average 1–12 grain size in the homogenized ingot is nearly 5 μm, along with smaller secondary grains. In the early stages of ball milling, the sharp decrease in powder size was due to the easy breakage of grain boundaries. The multi-grain powders were milled to the single-grain powders. However, as the milling continued, the powders, now consisting of single grain, were more resistant to breakage by ball milling. The break of 1–12 grains during prolonged milling accumulated stress.

Then, the hysteresis loops of the powders, depending on the ball milling time, were measured using VSM, as shown in Figure 2b,c. The powders were magnetically aligned under the magnetic field of 25 kOe. Figure 2d summarizes the values of *M_r_*/*M_s_* and *H_c_* calculated using the hysteresis loops. *M_r_*/*M_s_* and *H_c_* increased first shapely and then slowly because of the tendency of the powder size. The high value of *M_r_*/*M_s_* in the BM7-50 powders indicated that most powders were single-grain particles. Since the powder size became finer, the number of single-grain particles and the smaller grain size improved *M_r_*/*M_s_* and *H_c_*.

### 3.2. Microstructure and Magnetic Properties of Heat-Treated Bulks Influenced by Ball Milling Time

**Figure 3:**
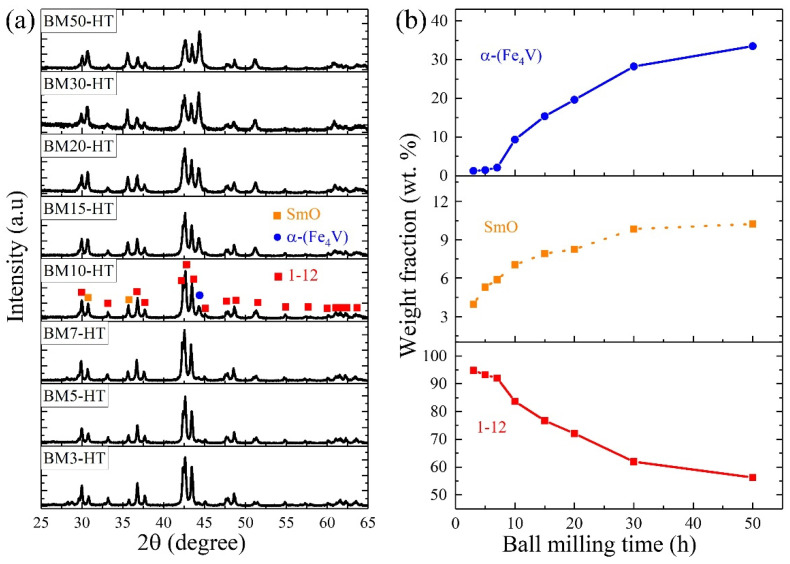
XRD patterns (**a**) and phase fractions (**b**) of the heat-treated bulks BM3-HT–BM50-HT. The fractions of α-(Fe_4_V), SmO, and 1–12 phases were summarized from the refinement of XRD patterns calculated by the FullProf program.

After preparing powders of different sizes, the green bodies pressed from these powders were heat-treated to obtain the bulk magnets. Figure 3a shows the XRD patterns of the heat-treated bulks. The bulks included the main 1–12 phase, along with secondary SmO and α-(Fe_4_V) phases. The intensity of the peaks indicates that the amounts of the SmO and α-(Fe_4_V) phases significantly increased as the ball milling time extended. In order to clearly explain the changes in each phase with different ball milling times, the XRD refinements were calculated and summarized in Figure S3 and Figure 3b. The SmO phase increased rapidly with short ball milling times and continued to increase at a slower rate beyond 10 h. The trend of the SmO phase change was similar to that of the powder size. From Figure 1 and Figure 2c, longer ball milling times resulted in finer particle sizes, leading to higher surface energy in the powders. Consequently, heavy oxidation occurred during the pressing and heat-treatment processes. The significant amount of SmO phase consumed free Sm, reducing the availability of Sm to form the Sm-rich GBP and the 1–12 phase. Conversely, the content of the α-(Fe_4_V) phase exhibited the opposite trend. It remained at a low level in the bulks from BM3-HT to BM7-HT but increased sharply from the BM10-HT sample and became a dominant phase with longer milling times. According to the analysis for the ball-milled powders, the accumulated stress in the powders decreased the stabilization of the ThMn_12_ crystal structure, which is the main reason for the significant presence of the α-(Fe_4_V) phase when ball milling times were too long.

**Figure 4:**
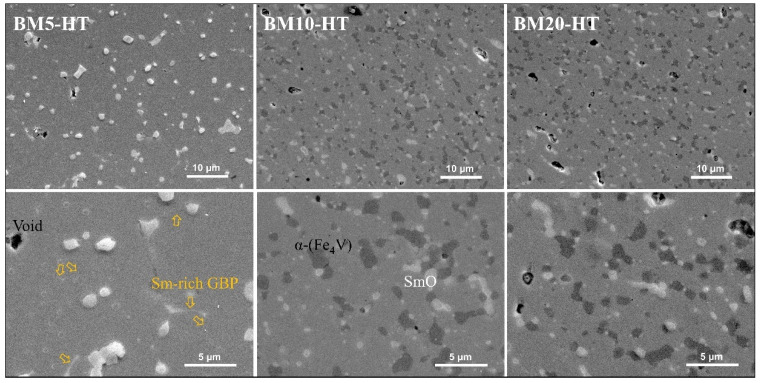
FE-SEM images of the heat-treated bulks fabricated from the BM5, BM10, and BM20 powders (BM5-HT, BM10-HT, BM20-HT). The heat-treated bulks include three phases: 1–12 (gray), α-(Fe_4_V) (black), and SmO (white). The yellow arrow shows the Sm-rich GBP (white color).

In order to investigate the microstructure of the heat-treated bulks in-depth, we examined the phase distribution using FE-SEM measurements. Figure 4 shows the FE-SEM images of the BM5-HT, BM10-HT, and BM20-HT bulks. In these images, three distinct phases can be observed, each with different colors. The main gray area represents the 1–12 phase. Most of the white areas are the SmO phase, with some regions being the Sm-rich phase on the 1–12 grain boundaries, as indicated by the yellow arrows. The black areas correspond to the α-(Fe_4_V) phase, which increased with longer ball milling times. It is noteworthy that the α-(Fe_4_V) phase appeared at the grain boundaries, and the number of small α-(Fe_4_V) grains increased instead of their growth as ball milling time extended. This indicates that in bulks made from long-time ball-milled powders, for example, BM10-HT and BM20-HT bulks, the Sm-rich GBP was replaced by small α-(Fe_4_V) grains. However, due to the strong ferromagnetic properties of the α-(Fe_4_V) phase, achieving effective magnetic decoupling of the 1–12 grains is challenging [20].

**Figure 5:**
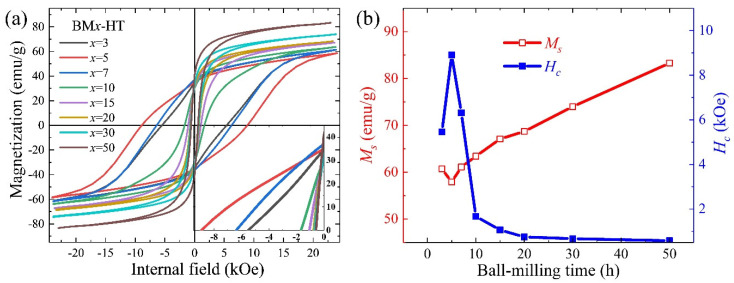
Demagnetization-corrected hysteresis loops (**a**) and the tendency of *M_s_* and *H_c_* (**b**) of the heat-treated bulks (BM3-HT–BM50-HT) depending on the ball milling time. *M_s_* and *H_c_* were calculated by the hysteresis loops.

Figure 5a shows the demagnetization-corrected hysteresis loops of the heat-treated bulks, depending on the ball milling time. The summarized data is presented in Figure 5b. The magnetic properties were strongly influenced by the ball-milled powder size. According to reference and our previous works [19,21], the enhanced *H_c_* resulted from the formation of the non-ferromagnetic Sm-rich GBP, which can promote magnetic decoupling of the 1–12 grains and hinder the domain wall motion. In relation to the XRD patterns in Figure 3 and SEM images in Figure 4, only the BM3-HT, BM5-HT, and BM7-HT bulks, which contained few α-(Fe_4_V) and SmO phases, exhibited high *H_c_*. As the ball milling time increased, heavy oxidation and decomposition of the 1–12 phase during the pressing and heat treatment processes led to the disappearance of the Sm-rich GBP. Consequently, *H_c_* sharply decreased due to the presence of a significant amount of α-(Fe_4_V) phase at the grain boundaries. Correspondingly, *M_s_* increased with the content of the α-(Fe_4_V) phase in the BM10-HT–BM50-HT samples.

### 3.3. Anisotropic Bulks Modified by the Pressure

In Section 3.2, we successfully fabricated isotropic bulk magnets with a high *H_c_*. However, due to their low magnetization and the low squareness of the hysteresis loops, (*BH*)*_max_* of the isotropic samples was very low, reaching only 2.15 MGOe in the BM5-HT sample, which had the highest *H_c_*. Therefore, we attempted to fabricate anisotropic bulks to improve (*BH*)*_max_*.

**Figure 6:**
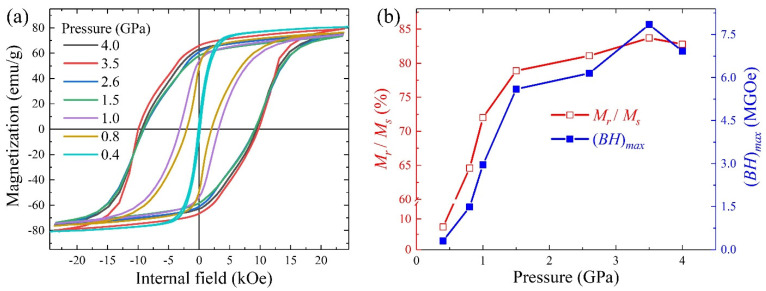
Demagnetization-corrected hysteresis loops (**a**), values of *M_r_*/*M_s_,* and (*BH*)*_max_* (**b**) of the anisotropic bulks depending on the pressure (AP-04–AP-40). The value of *M_r_*/*M_s_* represents the orientation level. The values of *M_s_*, *M_r_*, and (*BH*)*_max_* are shown in Table S1 in the Supplementary File.

Figure 6a shows the demagnetization-corrected hysteresis loops of the anisotropic bulks AP-04–AP-40, depending on the pressures applied during the high-pressure process. The detailed data are shown in Table S1 of the Supplementary File. High pressure strongly influences the microstructure and magnetic properties. *H_c_* of the AP-04 sample pressed under 0.4 GPa is only 0.13 kOe, characteristic of a near-soft magnet. As the pressure increased to above 1.5 GPa, *H_c_* remained between 9 and 10 kOe, with the highest value being 9.86 kOe in the AP-35 sample. It is evident that the *H_c_* of anisotropic bulks was higher than that of the isotropic one, which was 8.91 kOe. Additionally, *M_s_* at 23 kOe increased significantly from 59 emu/g to 73–79 emu/g, primarily due to the incomplete magnetic saturation in the isotropic bulks. Figure 6b summarizes the calculated value of *M_r_*/*M_s_* and (*BH*)*_max_* based on the hysteresis loops in Figure 6a. The value of *M_r_*/*M_s_* indicates the level of orientation in the anisotropic bulks. Higher pressure improved the orientation of the bulks up to 3.5 GPa. Beyond this point, at 4.0 GPa, the orientation decreased slightly. It is well known that high pressure leads to the significant deformation of green bodies. In our work, the pressure direction was perpendicular to the orientation direction, causing part deformation parallel to the orientation direction. Therefore, orientation improved with pressure up to 3.5 GPa, but excessive deformation at 4.0 GPa slightly reduced orientation. The highest *M_r_*/*M_s_* value, 83.7%, was achieved in the AP-35 bulk. High orientation is beneficial for (*BH*)*_max_*. In the anisotropic bulks, the highest (*BH*)*_max_* of 7.85 MGOe was achieved in the AP-35 bulk by the highest *M_s_*, *M_r_*/*M_s_*, and *H_c_*.

**Figure 7:**
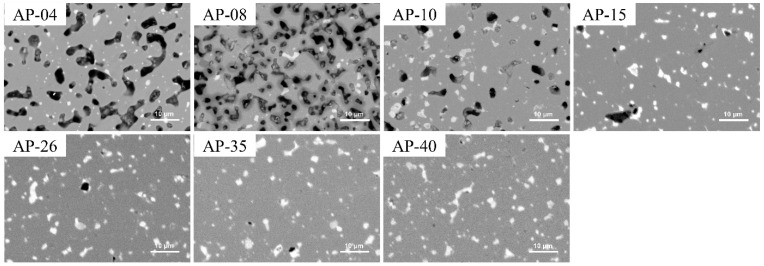
BSE-SEM images of the anisotropic heat-treated bulks (AP-04–AP-40) to check the density and phase influenced by the pressure. The black part represents the void.

Following the VSM measurements in Figure 6, the magnetic properties of the anisotropic bulks were studied in detail. Subsequently, the microstructure and phase were examined to explain the origin of these magnetic properties. Figure 7 shows the BSE-SEM images of anisotropic bulks pressed under different pressures. A large number of voids were present in the AP-04 and AP-08 bulk due to the low pressure. The 1–12 grains were either isolated or directly connected with each other. The low *H_c_* was attributed to the absence of the Sm-rich GBP. As the pressure increased, both the size and number of voids in the heat-treated bulks decreased. When the pressure was above 1.5 GPa, the white GBP, identified as the Sm-rich Fe-V-lean phase, became distinguishable among the main 1–12 grains. The number and area of voids significantly decreased with increasing pressure. This observation correlates with the density measurements of the bulks, taken using the Archimedes method. The densities measured were 7.456, 7.538, 7.545, 7.579, 7.674, 7.709, and 7.716 g/cm³ for the AP-04–AP-40 bulks, respectively. Higher pressure during the pressing process resulted in higher density, which in turn improved (*BH*)*_max_*.

**Figure 8:**
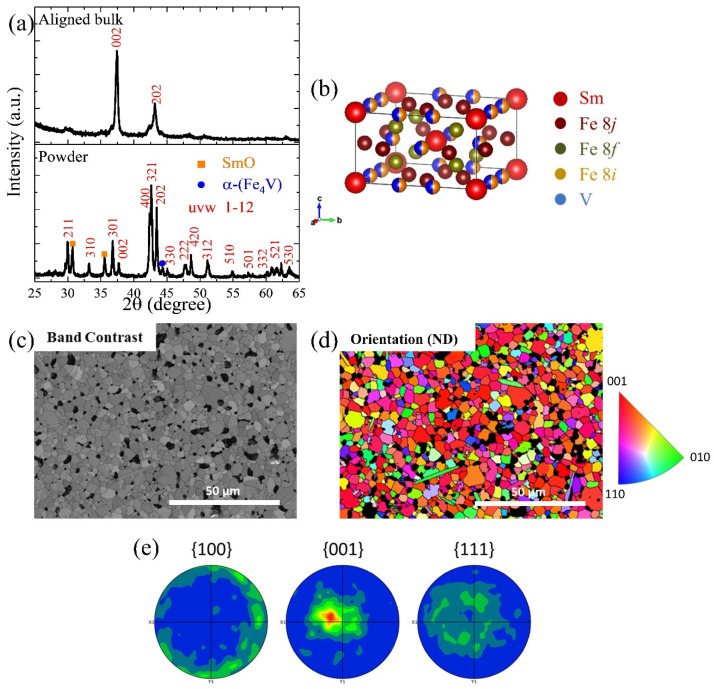
(**a**) XRD patterns of the sample AP-35, including bulk and ground powders; (**b**) crystal structure of the composition of SmFe_10_V_2_; (**c**) band contrast map; (**d**) inverse pole figure (IPF) map in the AP-35 sample’s surface normal (ND), in which the measuring face is perpendicular to the aligned direction; (**e**) polar figures of three different directions, calculated based on the EBSD image in (**d**).

Figure 8a shows the XRD patterns of the anisotropic bulk AP-35, which is pressed under 3.5 GPa. The upper pattern was measured from the bulk in the direction perpendicular to the alignment direction, while the lower pattern was measured from powders ground from the same bulk. The sample includes the main 1–12 phase, SmO, and a small amount of α-(Fe_4_V) phase, similar to the isotropic sample. As seen in the bulk XRD pattern, the main aligned direction is <002>, which corresponds to the easy magnetic alignment direction and the shortest *c*-axis in the crystal structure of Figure 8b. However, there is also a secondary alignment direction, <202>, rotated 45° from the main direction. To further investigate the orientation of each grain in the bulks, EBSD images were taken in the oriented direction, as shown in Figure 8c,d. The measurement surface normal was perpendicular to the oriented direction of the AP-35 bulk. The mean band contrast is 120.26. During the measurement, only the SmFe_10_V_2_ structure was loaded for indexing. Therefore, the black areas represent secondary phases and voids in the image. Most of the 1–12 grains appear red or in similar colors, indicating that the majority of the grains were aligned in a similar direction of <001>. As shown in Figure 8e, the polar figures in three different directions were calculated based on the EBSD image, confirming that the main orientation direction is parallel to <001>. Some grains were oriented in similar directions due to insufficient alignment, corroborating the XRD pattern results.

Although we successfully fabricated anisotropic bulks with high *H_c_*, there are some imperfections. The main issue is the defective squareness of the hysteresis loops, which held back the further improvement of (*BH*)*_max_*. Several factors contribute to these phenomena. One issue is that the external magnetic field was not strong enough to align all the powders in the same direction, as it needed to overcome the significant repulsive forces between the powders. The presence of a secondary alignment direction, detected in the XRD patterns in Figure 8a, proved this problem. Another possible reason is that not all powders were single-grain particles. Although the hydrogen decrepitation process weakened the grain boundaries, some small powders with multiple grains remained during the short-time ball milling process. These multi-grain powders compromised the level of orientation during the pressing and heat treatment processes. According to the FE-SEM images in Figure 4, the Sm-rich GBP was detected in the heat-treated bulks. However, it was not uniformly distributed among all grains. Some 1–12 grains lacked the white GBP due to inadequate diffusion during the 30 min heat treatment. To address these problems, we propose the following approaches:

1. Prevent Oxidation: The primary goal is to find a method to prevent heavy oxidation during the pressing and heat treatment processes. This would allow the use of finer powders to improve the alignment level and allocate more Sm to form the Sm-rich GBP instead of the SmO*_x_* phase.
2. Minimize Sm Evaporation: Research methods to reduce Sm evaporation during heat treatment. Ensuring a uniform GBP formation over a longer heat treatment period is essential, although this typically risks the decomposition of the 1–12 phase due to significant Sm evaporation.

By addressing these issues, the magnetic properties and performance of the anisotropic bulks can be further enhanced.

## 4. Conclusions

In this study, we investigated the influence of ball milling time on the microstructure and magnetic properties of the SmFe_10_V_2_ compound. As the powder size decreased, heavy oxidation, which consumed a significant amount of Sm, and increased strain led to the decomposition of the 1–12 phase. Anisotropic bulks were successfully fabricated by pressing and heat-treating the ball-milled powders. The maximum (*BH*)*_max_* of 7.85 MGOe was 3.7 times that of the isotropic bulk, which is attributed to the significantly improved *M_r_*, the squareness of the hysteresis loop, and a slightly enhanced *H_c_*. The use of a high pressure of 3.5 GPa proved beneficial in achieving high density and optimizing the alignment of the powders. This work modified the low-cost fabrication method using the ball milling process, revitalizing the development of ThMn_12_-type SmFe_12_-based anisotropic magnets.

## Figures and Tables

**Table 1:** Samples depending on the ball milling times (Group 1) and pressure (Group 2).

Group 1: Ball milling time dependence								
Ball milling time (h)	3	5	7	10	15	20	30	50
Ball-milled powders	BM3	BM5	Bm7	BM10	BM15	BM20	BM30	BM50
Heat-treated bulks (non-orientation)	BM3-HT	BM5-HT	BM7-HT	BM10-HT	BM15-HT	BM20-HT	BM30-HT	BM50-HT
Group 2: Pressure dependence (BM5 powders)								
Pressure (GPa)	0.4	0.8	1.0	1.5	2.6	3.5	4.0	
Heat-treated bulks (orientation)	AP-04	AP-08	AP-10	AP-15	AP-26	AP-35	AP-40	

## Data Availability

Data are contained within the article and Supplementary Materials.

## Supplementary Materials

The following supporting information can be downloaded at: https://www.mdpi.com/article/10.3390/nano14161329/s1, Figure S1: The powder size distribution of the ball-milled powders includes the cumulative distribution and density distribution, (a) BM3, (b) BM5, (c) BM7, (d) BM10, (e) BM15, (f) BM20, (g) BM30, (h) BM50; Figure S2: BSE-SEM images of the homogenized ingot (the right half is the original bulk; the left half is the corroded bulk by 2 % Nital); Figure S3: Rietveld refined X-ray diffraction patterns of the bulks BMx-HT (x = 3–50) by the FullProf program; Table S1: Demagnetization-corrected factors (Dz), Ms, Mr, the value of Mr/Ms, density, Hc, and (BH)max of the anisotropic bulks depending on the pressure (AP04–AP40).

## References

[1] Gabay A.M., Hadjipanayis G.C. (2018). Recent Developments in RFe_12_-Type Compounds for Permanent Magnets. Scr. Mater..

[2] Hadjipanayis G.C., Gabay A.M., Schönhöbel A.M., Martín-Cid A., Barandiaran J.M., Niarchos D. (2020). ThMn_12_-Type Alloys for Permanent Magnets. Engineering.

[3] Hirayama Y., Takahashi Y.K., Hirosawa S., Hono K. (2017). Intrinsic Hard Magnetic Properties of Sm(Fe_1−x_Co_x_)_12_ Compound with the ThMn_12_ Structure. Scr. Mater..

[4] Dirba I., Harashima Y., Sepehri-Amin H., Ohkubo T., Miyake T., Hirosawa S., Hono K. (2020). Thermal Decomposition of ThMn_12_-Type Phase and Its Optimum Stabilizing Elements in SmFe_12_-Based Alloys. J. Alloys Compd..

[5] Makurenkova A., Ogawa D., Tozman P., Okamoto S., Nikitin S., Hirosawa S., Hono K., Takahashi Y.K. (2021). Intrinsic Hard Magnetic Properties of Sm(Fe,Co)_12−x_Ti_x_ Compound with ThMn_12_ Structure. J. Alloys Compd..

[6] Tang X., Li J., Srinithi A.K., Sepehri-Amin H., Ohkubo T., Hono K. (2021). Role of V on the Coercivity of SmFe_12_-Based Melt-Spun Ribbons Revealed by Machine Learning and Microstructure Characterizations. Scr. Mater..

[7] Sun H., Otani Y., Coey J.M.D., Meekison C.D., Jakubovics J.P. (1990). Coercivity and Microstructure of Melt-spun Sm(Fe_11_Ti). J. Appl. Phys..

[8] Liu Z., Liu Z., Wu H., Zhu C., Cheng W., Cao S., Luo H., Wu L., Chen R., Xia W. (2023). Mechanism of Ti-Rich Grain Boundary Phase Formation and Coercivity Reinforcement in Sm(Fe_0.8_Co_0.2_)_11_TiB_x_ Melt-Spun Ribbons. Scr. Mater..

[9] Qian H.-D., Lim J.T., Kim J.-W., Yang Y., Zhou T.H., Jeon H.K., Park J., Choi C.-J. (2022). Physical and Magnetic Properties of ThMn_12_-Type Sm(Fe_0.8_Co_0.2_)_10_Si_2_ Melt-Spun Ribbons. Metals.

[10] Qian H.-D., Lim J.T., Kim J.-W., Yang Y., Cho K.M., Park J., Choi C.-J. (2021). Phase Transformation and Magnetic Properties of Fully Dense Sm(Fe_0.8_Co_0.2_)_11_Ti Bulk Magnets. Scr. Mater..

[11] Zhao L., Li C., Zhang X., Bandaru S., Su K., Liu X., Zhou Q., Li L., Greneche J.-M., Jin J. (2020). Effects of Sm Content on the Phase Structure, Microstructure and Magnetic Properties of the Sm_x_Zr_0.2_(Fe_0.8_Co_0.2_)_11.5_Ti_0.5_ (x = 0.8−1.4) Alloys. J. Alloys Compd..

[12] Tozman P., Fukazawa T., Ogawa D., Sepehri-Amin H., Bolyachkin A., Miyake T., Hirosawa S., Hono K., Takahashi Y.K. (2022). Peculiar Behavior of V on the Curie Temperature and Anisotropy Field of SmFe_12−x_V_x_ Compounds. Acta Mater..

[13] Srinithi A.K., Sepehri-Amin H., Tang X., Tozman P., Li J., Zhang J., Kobayashi S., Ohkubo T., Nakamura T., Hono K. (2021). Phase Relations and Extrinsic Magnetic Properties of Sm–(Fe,Co)–Ti–(Ga)-Based Alloys for ThMn_12_-Type Permanent Magnets. J. Magn. Magn. Mater..

[14] Zhou T.H., Qian H.-D., Lim J.T., Jeon H.-K., Choi C.-J., Cho Y.-R., Park J. (2023). Effects of Ti–V Pair Substitution and Grain Boundary Modification on Physical and Magnetic Properties of Sm(Fe_0.8_Co_0.2_)_12_ Bulk Magnet. J. Alloys Compd..

[15] Schönhöbel A.M., Madugundo R., Gabay A.M., Barandiarán J.M., Hadjipanayis G.C. (2019). The Sm-Fe-V Based 1:12 Bulk Magnets. J. Alloys Compd..

[16] Schönhöbel A.M., Madugundo R., Barandiarán J.M., Hadjipanayis G.C., Palanisamy D., Schwarz T., Gault B., Raabe D., Skokov K., Gutfleisch O. (2020). Nanocrystalline Sm-Based 1:12 Magnets. Acta Mater..

[17] Zhang J.S., Tang X., Sepehri-Amin H., Srinithi A.K., Ohkubo T., Hono K. (2021). Origin of Coercivity in an Anisotropic Sm(Fe,Ti,V)_12_-Based Sintered Magnet. Acta Mater..

[18] Otsuka K., Kamata M., Nomura T., Iida H., Nakamura H. (2021). Coercivities of Sm–Fe–M Sintered Magnets with ThMn_12_-Type Structure (M = Ti, V). Mater. Trans..

[19] Zhou T.H., Song Y., Zhang B., Zheng X., Choi C.-J., Cho Y.-R., Park J. (2024). Enhancing Coercivity through Grain Boundary Phase Modification in Sm_x_Fe_10_V_2_. J. Mater. Res. Technol..

[20] Palanisamy D., Ener S., Maccari F., Schäfer L., Skokov K.P., Gutfleisch O., Raabe D., Gault B. (2020). Grain Boundary Segregation, Phase Formation, and Their Influence on the Coercivity of Rapidly Solidified SmFe_11_Ti Hard Magnetic Alloys. Phys. Rev. Mater..

[21] Srinithi A.K., Tang X., Sepehri-Amin H., Zhang J., Ohkubo T., Hono K. (2023). High-Coercivity SmFe_12_-Based Anisotropic Sintered Magnets by Cu Addition. Acta Mater..

