# Supplementary material for: Anisotropic SmFe10V2 Bulk Magnets with Enhanced Coercivity via Ball Milling Process
Source: Nanomaterials (Basel). 2024 Aug 8;14(16):1329. doi: 10.3390/nano14161329 (PMC11357663; doi:10.3390/nano14161329)
Supplement: Supplementary file 1 [file nanomaterials-14-01329-s001.zip › nanomaterials-3102521-supplementary.pdf]

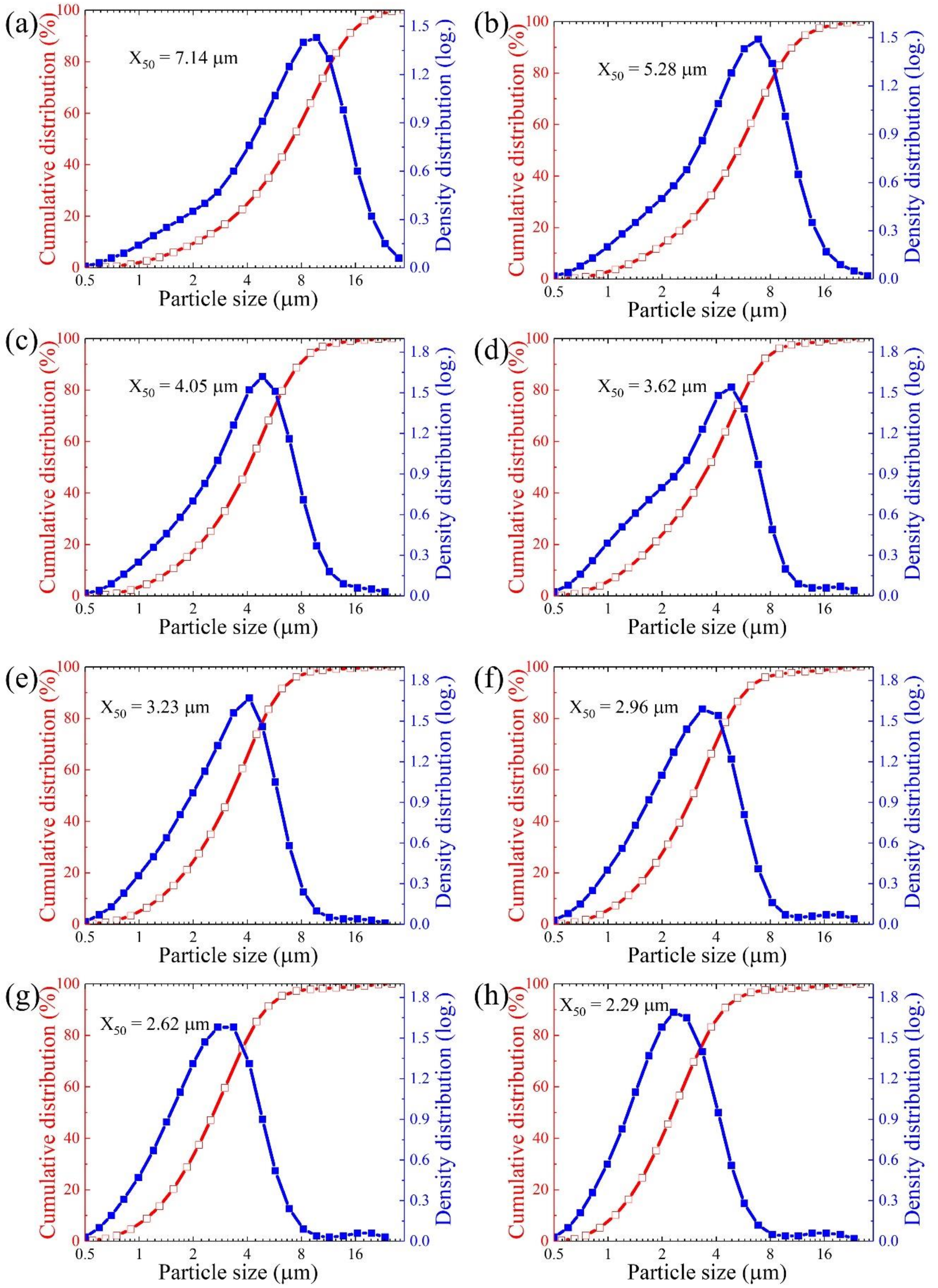

Fig. S1. The powder size distribution of the ball-milled powders includes the cumulative distribution and density distribution, (a) BM3, (b) BM5, (c) BM7, (d) BM10, (e) BM15, (f) BM20, (g) BM30, (h) BM50.

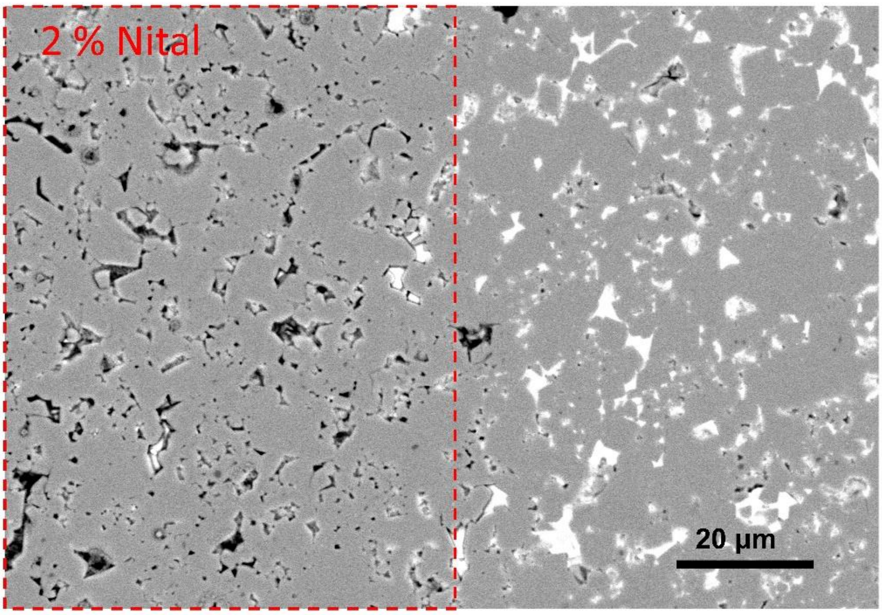

Fig. S2. BSE-SEM images of the homogenized ingot (the right half is the original bulk; the left half is the corroded bulk by 2 % Nital).

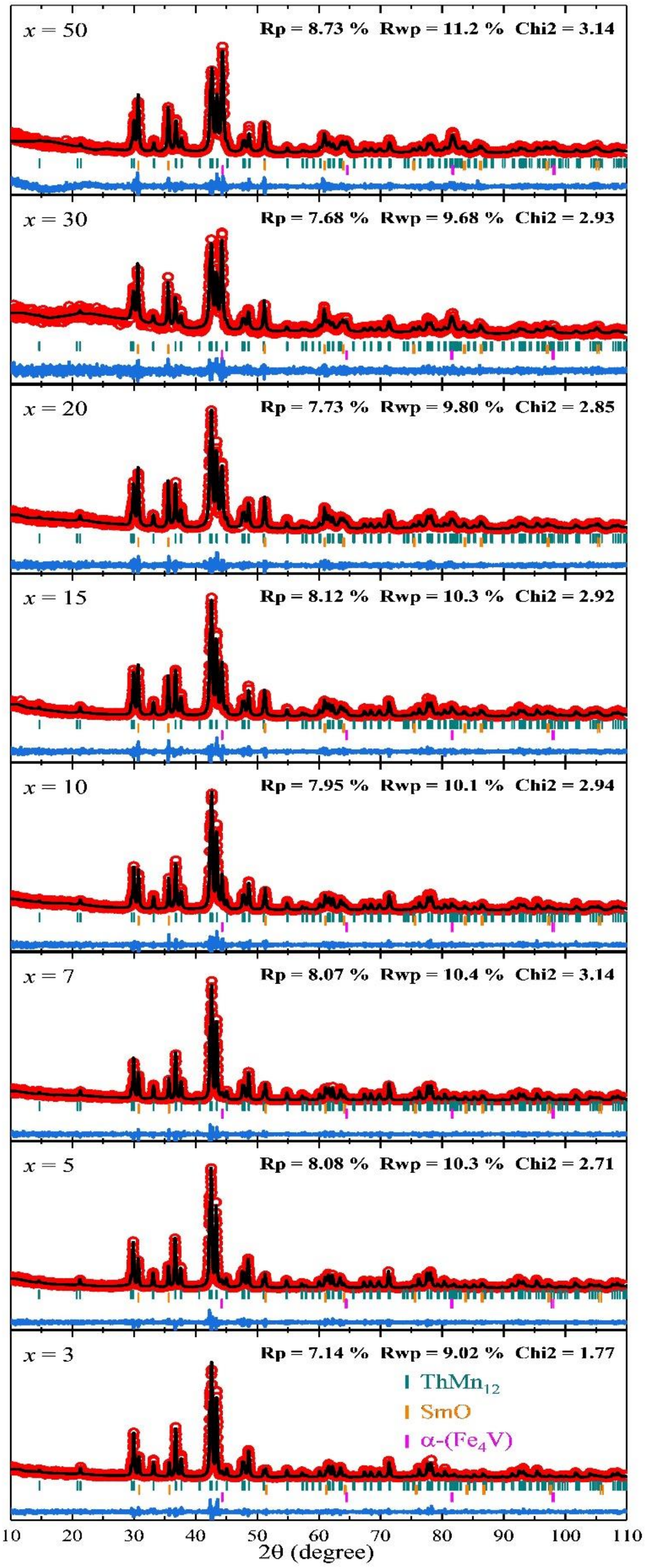

Fig. S3. Rietveld refined X-ray diffraction patterns of the bulks BM*x*-HT (*x* = 3-50) by the FullProf program.

Table S1. Demagnetization-corrected factors (D_z), M_s, M_r, the value of M_r / M_s, density, H_c, and $(BH)_{max}$ of the anisotropic bulks depending on the pressure (AP04 - AP40).

Pressure (GPa)	D_z	M_s at 23 kOe (emu/g)	M_r (emu/g)	M_r / M_s (%)	ρ (g/cm^3)	H_c (kOe)	$(BH)_{max}$ (MGOe)
4.0	0.20	75.8	62.9	82.8	7.716	9.40	6.92
3.5	0.16	79.5	66.6	83.7	7.709	9.86	7.85
2.6	0.19	75.0	61.0	81.1	7.674	9.09	6.15
1.5	0.22	73.6	58.3	78.9	7.579	9.15	5.60
1.0	0.20	74.3	53.5	72.0	7.545	3.19	2.96
0.8	0.18	76.0	49.1	64.6	7.538	2.02	1.49
0.4	0.27	81.0	6.00	7.41	7.456	0.137	0.306
